# Some plasma biomarkers of residual feed intake in beef cattle remain consistent regardless of intake level

**DOI:** 10.1038/s41598-024-59253-7

**Published:** 2024-04-12

**Authors:** G. Cantalapiedra-Hijar, K. Nedelkov, P. Crosson, M. McGee

**Affiliations:** 1 Université Clermont Auvergne, INRAE, VetAgro Sup, UMR Herbivores, 63122 St-Genès-Champanelle, France; 2 Faculty of Veterinary Medicine, Trakia University, Stara Zagora, 6000 Bulgaria; 3 Teagasc, Animal & Grassland Research and Innovation Centre, Grange, Dunsany, Co. Meath, Ireland

**Keywords:** Animal physiology, Metabolic engineering

## Abstract

This study investigated whether plasma biomarkers of residual feed intake (RFI), identified under ad libitum feeding conditions in beef cattle, remained consistent during feed restriction. Sixty Charolais crossbred young bulls were divided into two groups for a crossover study. Group A was initially fed ad libitum (first test) and then restricted (second test) on the same diet, while Group B experienced the opposite sequence. Blood samples were collected from the 12 most divergent RFI animals in each group at the end of the first test and again after the second test. 12 plasma variables consistently increased, while three consistently decreased during feed restriction (FDR < 0.05). Only two metabolites, α-aminoadipic acid for Group A and 5-aminovaleric acid for Group B, were associated with RFI independent of feed intake level (FDR < 0.05), demonstrating moderate-to-high repeatability across feeding levels (intraclass correlation coefficient ≥ 0.59). Notably, both metabolites belong to the same metabolic pathway: lysine degradation. These metabolites consistently correlated with RFI, irrespective of fluctuations in feed intake, indicating a connection to individual metabolic processes influencing feed efficiency. These findings suggest that a portion of RFI phenotypic variance is inherent to an individual’s metabolic efficiency beyond variations in feed intake.

## Introduction

Improving animal feed efficiency (FE) can significantly contribute to enhancing the sustainability of the beef cattle sector^1^. In addition to established nutritional strategies aimed at improving the average FE of co-reared animal groups^2,3^, there is, within the same breed, significant variability in FE among individual animals reared under identical conditions^4^. This variability in FE among animals, coupled with its moderate heritability, provides an opportunity for the genetic selection of superior animals for this trait^5^, a practice currently employed in cattle breeding programs^6^. In addition to genetic selection, understanding and forecasting the interindividual variability in FE could form the basis for innovative individual animal-centered feeding strategies or precision nutrition approaches^4,7^. Precision feeding can further improve resource use efficiency, reduce animal excretions to the environment, and enhance the financial viability of livestock farming^8^. Indeed, as highlighted by Tedeschi et al.^7^, the identification of animals with superior FE is among the most influential factors towards achieving long-term livestock sustainability.

Among the metrics used for assessing beef cattle FE, residual feed intake (RFI) stands out as one of the most widely employed indices within animal breeding^9^. RFI enables the identification of individuals with lower feed intake while maintaining similar performance levels (i.e., superior FE)^10^. Nevertheless, due to the substantial cost and time associated with determining RFI in beef cattle, several blood biomarkers that correlate with the biological mechanisms underlying RFI have been proposed as alternative cost-effective tools for identifying efficient (i.e., low-RFI) cattle^11,12^. In this regard, literature reviews^4,13^ highlighted that the primary biological mechanisms driving RFI appear to be intrinsically connected to animal metabolism. However, whether these metabolic regulations play a causal role or are merely associated with RFI due to covariance with feed intake remains uncertain. For instance, multiple studies have suggested that the plasma concentration of branched-chain amino acids, having signalling roles in key metabolic pathways such as protein and lipid synthesis, could serve as potential biomarkers of RFI in beef cattle^11,14–17^. Likewise, a meta-analysis of genome-wide association studies conducted on beef cattle concluded that the only metabolic pathway genetically linked to RFI was the degradation pathway of branched-chain amino acids^18^. However, it is important to note that the plasma concentration of branched-chain amino acids in ruminants, much like other nutrients, is significantly influenced by feed protein intake and nutrient flow to the duodenum^19^. This confounding effect makes it challenging to determine whether these biomarkers capture processes that render individuals more metabolically efficient, or conversely, mirror reduced amino acid absorption resulting from the lower feed protein intake associated with efficient RFI phenotypes. Gaining insight into the manner in which blood metabolites are linked to phenotypes becomes pivotal before proposing them as robust biomarkers for assisting genetic selection and precision feeding. In other words, for the successful application of RFI biomarkers, it is crucial to comprehend the extent to which these blood metabolites are correlated with animal feed intake (feeding level) and host-related physiological differences independent of nutrient intake^20^, such that metabolic feed efficiency is enhanced across contrasting conditions encompassing both feed abundance and scarcity.

We propose the hypothesis that numerous previously identified candidate biomarkers of RFI are associated with this phenotype primarily due to their blood or plasma concentrations covarying with nutrient intake. In contrast, certain other metabolites, such as branched-chain amino acids, may experience changes in their concentration when nutrient intake is altered, yet still retain a component linked to the animal’s host physiology that these biomarkers can capture regardless of fluctuations in feeding level. Consequently, the aim of this study was to ascertain whether plasma biomarkers of RFI, identified under controlled ad libitum feeding conditions, remain consistent when the same animals are offered the same diet but at a restricted and identical feeding level.

## Results

Mean metabolic body weight (BW) emerged as the sole consistent and significant predictor across all four RFI models (Supplementary Table S2), while average daily gain (ADG) was only significant in the models involving animals with ad libitum feeding. Nearly all the observed variability in dry matter (DM) intake within feed restriction conditions was explained through predictors in the RFI model (r2 = 0.98 and 0.99), leading to minor fluctuations in RFI values, as indicated by their coefficients of variation (CV) of 0.59 and 0.64, respectively. Conversely, the RFI models accounted for only 81% and 51% of the explained variance within ad libitum conditions for the first and second test periods, respectively, resulting in RFI value CVs of 3.85% and 5.08%, respectively. As explained further, the RFI phenotype measured in ad libitum conditions was maintained as identical during feed restriction to assess whether the identified RFI biomarkers were still linked to the RFI phenotype when animals were fed at the same feeding level.

Animal performance per test period (first vs. second RFI test) and feeding level (ad-libitum vs. restriction) are presented in Table 1. There were statistically significant feeding level × test period interactions for DM intake (kg/day and kg/100 kg BW), start and end BW, mean metabolic BW (P < 0.001), ADG (P < 0.01) and feed conversion efficiency (FCE) (P < 0.05). The expected increase in DM intake in ad libitum compared to feed restriction exhibited a more pronounced effect during the first (0–70 days) compared to the second (78–148 days) RFI test (42% vs. 24%, respectively). By design, the start BW did not differ between the feeding level groups during Test 1. However, due to greater growth performance, the end BW in Test 1 with ad libitum feeding (Group A) was significantly greater (P < 0.05) compared to restricted feeding (Group B). Consequently, in Test 2, the initial BW of Group A animals (subjected to feed restriction) was also greater than those from Group B (subjected to ad libitum feeding).. End BW and mean metabolic BW were greater for the ad libitum group than for the restricted group in Test 1 but did not differ between the feeding level groups in Test 2. Daily live weight gain for the ad libitum group did not differ between the two tests, whereas for the restricted group, it was lower in Test 1 than in Test 2. Feed conversion efficiency for the ad-libitum groups was greater during Test 1 compared to Test 2, whereas there was no difference between tests for the restricted groups. No significant interactions (P > 0.05) between feeding level and test period were observed for changes in ultrasound fat and muscle depth measurements; however, these parameters were all influenced by feeding level (P < 0.001), with greater changes observed with ad libitum feeding compared to feed restriction. The test period impacted the change in muscle depth (P < 0.001) and indicated a trend in rib fat depth change (P = 0.07), with increases in depth more substantial during Test 1 compared to Test 2.

**Table 1:** Performance of growing-fattening bulls fed ad libitum and then restricted (Group A) or vice versa (Group B) during two consecutive 70-d feed efficiency tests.

	Test 1 (0–70 days)		Test 2 (78–148 days)		SEM	*P value*		
	Ad-libitum (Group A)	Restriction (Group B)	Ad-libitum (Group B)	Restriction (Group A)		Feeding level	Feeding period	FL × P
Number of animals	29	30	30	28				
Age, days	394	390	467	470	8.35	0.77	< 0.001	0.69
DM intake, kg/days	9.72^b^	6.84^d^	10.4^a^	8.44^c^	0.114	< 0.001	< 0.001	< 0.001
DM intake, kg/100 kg BW	1.91^a^	1.46^c^	1.78^b^	1.41^c^	0.015	< 0.001	< 0.001	< 0.001
Body weight, kg								
Start	451^c^	450^c^	527^b^	563^a^	4.38	< 0.001	< 0.001	< 0.001
End	562^b^	502^c^	632^a^	627^a^	5.36	< 0.001	< 0.001	< 0.001
Mean metabolic BW, kg	107^b^	102^c^	118^a^	120^a^	0.76	0.09	< 0.001	< 0.001
Average daily gain^1^, kg	1.59^a^	0.74^c^	1.49^a^	0.92^b^	0.0416	< 0.001	0.32	0.001
Fat depth change^2^, mm								
Rump	2.14	0.86	2.77	0.75	0.256	< 0.001	0.29	0.15
Lumbar	0.631	0.391	0.620	0.156	0.0925	< 0.001	0.19	0.23
Rib	1.51	0.410	1.12	0.212	0.161	< 0.001	0.07	0.54
Muscle depth change^2^, mm	11.1	6.67	6.06	0.59	0.878	< 0.001	< 0.001	0.54
Feed conversion efficiency, kg BW gain/kg DM intake	0.163^a^	0.107^c^	0.144^b^	0.109^c^	0.0039	< 0.001	0.017	0.010

^a-d^Mean values with different superscripts are significantly different (P < 0.05).

^1^Average daily live weight gain calculated as the difference between final and initial body weight divided by the duration (70 days).

^2^Calculated as the difference between depth values measured at the end minus those obtained at the beginning.

Overall animal performances from day 0–148 per group (A vs. B) are presented in Table 2. Bulls assigned to Group A (starting the first RFI test with ad libitum feeding and then transitioning to restricted feeding) had a greater overall DM intake (P = 0.001) and similar overall ADG (P = 0.36), resulting in poorer FCE (P = 0.002) and RFI (P < 0.001) values compared to those assigned to Group B. Additionally, animals from Group A tended to have a lower overall change in rump fat depth (P = 0.08) and a higher kill-out proportion (P = 0.09). Slaughter weight, carcass weight and carcass conformation and fat scores did not differ (P ≥ 0.32) between animals in Groups A and B.

**Table 2:** Overall performance (0–148 days) of growing-fattening bulls fed ad libitum (0–70 days) and then restricted (78–148 days) (Group A) or vice versa (Group B).

	Group A (Ad-libitum → Restriction)	Group B (Restriction → Ad-libitum)	SEM	*P value*
Number of animals	28	30		
Body weight at slaughter^1^, kg	651	651	6.52	0.99
Carcass weight, kg	375	369	4.51	0.32
Kill-out proportion^2^, %	57.7	56.7	0.42	0.09
Carcass conformation score (1–15)^3^	10.0	10.0	0.24	1.00
Carcass fat score (1–15)^4^	6.11	6.23	0.23	0.70
Performance from 0 to 148 days				
Total DM intake, kg	9.06	8.59	0.100	0.001
Total average daily gain, kg	1.20	1.24	0.029	0.36
Feed conversion efficiency, kg BW gain/kg DM intake	0.132	0.144	0.0026	0.002
Residual feed intake^5^, kg/days	0.160	-0.150	0.057	< 0.001
Fat depth change^6^, mm				
Rump	2.92	3.63	0.286	0.08
Lumbar	0.779	1.01	0.105	0.12
Rib	1.73	1.52	0.194	0.46
Muscle depth change, mm	11.7	12.7	0.935	0.45

^1^Animals were slaughtered approximately 2 weeks after the end of the second feed efficiency test.

^2^Kill-out proportion: (Cold carcass weight/Body weight at slaughter) × 100.

^3^EU beef carcass classification scheme scale, where 1 represents the poorest and 15 the best conformation.

^4^EU beef carcass classification scheme scale, where 1 represents the leanest and 15 the fattest carcass.

^5^Residual feed intake calculated through the entire experimental period (0–148 days) with a model having significant DM intake predictors the block, pen and metabolic body weight (r2 = 0.64).

^6^Calculated as the difference between depth values measured at the end minus those obtained at the beginning.

Table 3 illustrates performance per test period for the 24 most divergent bulls for RFI (6 low-RFI and 6 high-RFI per feeding level), determined during the initial (0–70 days) RFI test. In Test 1, the low-RFI bulls in both Group A (ad-libitum) and Group B (restricted) did not differ (P > 0.05) in terms of DM intake (kg/day), mean metabolic BW, ADG, and FCE when compared to the high-RFI animals. In contrast, DM intake relative to BW was lower (P < 0.05) for the low- compared to the high-RFI animals in Group A, whereas there was no difference between the RFI groups in Group B. As expected, RFI values differed between the low-RFI and high-RFI groups (P < 0.001), with a mean difference of 0.99 kg DM/day for Group A (ad-libitum) and only 0.115 kg DM/day for Group B (restricted). In Test 2, the low- and high-RFI bulls in Group A (now restricted) did not differ in DM intake (kg/d or kg/100 kg BW), mean metabolic BW, ADG, FCE and RFI, whereas in Group B (now ad-libitum), the low-RFI bulls had a higher DM intake in kg/day or kg/100 kg BW and ADG (P < 0.05) and tended to have a greater FCE (P = 0.06) than the high-RFI animals. It is noteworthy to highlight that, although RFI values were less extreme in restricted feeding conditions compared to ad-libitum feeding for Group A, on average, they still exhibited negative values for low-RFI animals and positive ones for high-RFI values (P = 0.13). In contrast, feed-efficient animals (low-RFI) starting with feed restriction (Group B) showed, on average, positive values during ad-libitum feeding, and the reverse was observed for inefficient animals (P = 0.24).

**Table 3:** Performances of the top 24 growing-fattening bulls with divergent residual feed intake (RFI) values (12 in group A and 12 in group B) identified during the initial 0–70 days feed efficiency test and selected for plasma metabolomics analysis conducted in the first and second feed efficiency tests.

	First RFI test (0–70 days)							
	Group A—Ad-libitum				Group B—Restricted			
	Low-RFI	High-RFI	SEM	P value	Low-RFI	High-RFI	SEM	P value
Number of animals	6	6			6	6		
DM intake, kg/days	9.27	10.1	0.401	0.19	6.73	6.87	0.135	0.49
DM intake, kg/100 kg BW	1.80	1.98	0.055	0.04	1.46	1.46	0.004	0.42
Metabolic body weight, kg	105	107	1.5	0.37	101	101	1.5	0.90
Average daily gain, kg	1.61	1.54	0.136	0.72	0.64	0.69	0.073	0.63
FCE^1^, kg BW gain/kg DM intake	0.173	0.152	0.010	0.17	0.095	0.100	0.010	0.72
Residual feed intake, kg DM/days	− 0.56	0.44	0.077	< 0.001	-0.059	0.057	0.013	< 0.001

	Second RFI test (78–148 days)*							
	Group A—Restricted				Group B—Ad-libitum			
	Low-RFI	High-RFI	SEM	P value	Low-RFI	High-RFI	SEM	P value
Number of animals	6	6			6	6		
DM intake, kg/days	8.47	8.37	0.191	0.74	10.8	9.78	0.267	0.02
DM intake, kg/100 kg BW	1.41	1.42	0.005	0.16	1.86	1.72	0.041	0.04
Metabolic body weight, kg	119	121	2.0	0.50	118	116	2.0	0.41
Average daily gain, kg	0.93	0.91	0.076	0.91	1.66	1.31	0.086	0.02
FCE^1^, kg BW gain/kg DM intake	0.110	0.109	0.0085	0.96	0.153	0.133	0.0064	0.06
Residual feed intake, kg DM/days	− 0.027	0.015	0.018	0.13	0.140	-0.201	0.194	0.24

*Animals in the second RFI test kept the same RFI ranking (lowest vs highest) as in the first test. However, the RFI values shown in the table are the actual RFI values for those animals in the second RFI test.

^1^FCE, feed conversion efficiency.

Among the 74 plasma variables analysed in the same 24 RFI-divergent bulls across both test periods, 21 variables exhibited an increase (P < 0.05) in their concentration or values during feed restriction, while 12 variables decreased (P < 0.05; Table 4). Following FDR correction (FDR < 0.05), among the plasma variables that exhibited a significant increase in concentration or values, there were seven amino acids or related metabolites (1-methylhistidine, 3-methylhistidine, 5-aminovaleric acid, β-aminobutyric acid, betaine, sarcosine, and glycine), four fatty acids (docosahexaenoic, eicosapentaenoic, octadecenoic, and octadecadienoic acids), as well as non-esterified fatty acids and the natural abundance of^15^N. Conversely, following FDR corrections (FDR < 0.05), only β-alanine, hippuric acid, and β-hydroxybutyrate demonstrated a significant decrease during feed restriction. All the metabolites that demonstrated higher or lower concentrations/values during feed restriction exhibited consistent behaviour across test periods (i.e., no feeding level × period interaction; P > 0.05). Of these metabolites, the feeding period exclusively affected sarcosine, docosahexaenoic acid, eicosapentaenoic acid, and the natural abundance of ^15^N (all displaying higher concentrations or values in the first test period) following FDR corrections (FDR < 0.05). The repeatability of plasma metabolites or variables analysed in the same 24 RFI-divergent bulls across two feeding levels is detailed in Table 5. Six plasma variables exhibited ‘high’ repeatability (r > 0.70), while 24 exhibited ‘moderate’ repeatability (0.40 ≥ r ≥ 0.70) across feeding periods. Notably, 3-methylhistidine and alkaline phosphatase exhibited repeatability values ≥ 0.90 across feeding levels, indicating that individuals are consistently ranked for these two variables regardless of the feeding level and test period.

**Table 4:** Plasma metabolites (μM) and variables significantly (P < 0.05) affected by feeding level (FL) in most extreme RFI bulls fed ad libitum (0–70 days) and then restricted (78–148 days) (Group A) or vice versa (Group B).

	Test 1 (0–70 days)		Test 2 (78–148 days)		Fold change	SEM	*P value*		
	Ad-libitum (Group A)	Restricted (Group B)	Ad-libitum (Group B)	Restricted (Group A)			Feeding level	Feeding period	FL × P
Number of animals	12	12	12	12					
Increased during restriction									
Amino acid related									
1-Methyl-His	6.53	7.83	6.51	8.38	1.24	0.460	< 0.0001*	0.256	0.642
3-Methyl-His	5.40	6.00	5.32	6.17	1.14	0.461	0.001*	0.739	0.843
5-Aminovaleric acid	1.19	1.78	1.02	1.41	1.44	0.153	0.0001*	0.012	0.595
β-aminobutyric acid	0.114	0.134	0.092	0.115	1.21	0.0091	0.0001*	0.002	0.898
Betaine	117	138	96.4	118	1.20	7.72	< 0.0001*	< 0.0001	0.972
Creatinine	139	152	131	143	1.09	7.71	0.009	0.062	0.974
Sarcosine	1.99	2.68	1.54	1.90	1.30	0.191	0.003*	0.0007*	0.467
Amino acids									
Glycine	409	528	356	482	1.32	23.7	< 0.0001*	0.032	0.894
Histidine	53.1	66.2	53.4	61.3	1.20	4.12	0.006	0.519	0.587
Proline	66.0	75.4	66.9	68.3	1.08	2.85	0.045	0.230	0.218
Serine	91.9	138	71.2	98.4	1.45	7.73	< 0.0001*	0.0002*	0.277
Bile acids									
Cholic acid	7.30	9.01	5.90	12.7	1.64	1.66	0.013	0.467	0.159
Chenodeoxycholic acid	0.386	0.449	0.259	0.558	1.56	0.086	0.043	0.915	0.194
Deoxycholic acid	0.868	0.983	0.634	2.089	2.05	0.358	0.038	0.233	0.078
Fatty acids									
Nonesterified fatty acids	88.9	143	105	266	2.14	26.0	0.0004*	0.016	0.059
Arachidonic acid	1.123	1.386	0.686	1.056	1.35	0.167	0.027	0.009	0.786
Docosahexaenoic acid	0.847	1.173	0.530	0.802	1.43	0.0871	0.0002*	0.0009*	0.811
Eicosapentaenoic acid	0.251	0.316	0.144	0.262	1.46	0.0241	0.0003*	0.001*	0.336
Octadecenoic acid	46.5	61.1	40.5	92.3	1.76	8.58	0.0005*	0.108	0.047
Octadecadienoic acid	4.84	6.59	5.13	9.84	1.65	0.886	0.0011*	0.050	0.113
Natural isotope abondance, ‰									
δ^15^N	5.64	5.88	5.29	5.61	1.02	0.095	0.0006*	0.0002*	0.768
Decreased during restriction									
Amino acid related									
Carnosine	20.7	18.0	19.7	19.4	0.93	1.29	0.018	0.678	0.495
Homoarginine	3.73	3.54	3.44	2.77	0.88	0.298	0.016	0.004	0.543
Amino acids									
Glutamine	406	382	423	389	0.93	14.3	0.032	0.353	0.741
Glutamate	88.3	78.7	79.0	70.8	0.89	6.23	0.032	0.039	0.929
Isoleucine	103	93.3	108	98.4	0.91	3.89	0.007	0.138	0.972
Tryptophane	43.4	37.3	43.2	42.0	0.92	2.52	0.036	0.177	0.439
Tyrosine	52.0	39.4	53.4	48.8	0.84	2.98	0.001	0.025	0.280
Biogenic amines									
β-alanine	3.95	2.80	3.49	3.00	0.78	0.291	0.008*	0.660	0.283
Carboxilic acids									
Hippuric acid	75.4	46.5	84.8	59.3	0.66	5.80	0.0001*	0.069	0.774
Indoles									
Indolepropionic acid	4.26	3.15	2.74	2.03	0.74	0.571	0.027	0.0024*	0.780
Ketone bodies									
β-hydroxybutyrate	389	273	445	308	1.44	22.8	< 0.0001*	0.060	0.656
Hormones, ng/mL									
IGF-1	528	402	502	458	1.20	46.5	0.02	0.65	0.49

*P values with a star are also FDR < 0.05.

**Table 5:** Repeatable plasma metabolites and parameters in growing-fattening bulls fed ad libitum (0–70 days) and then restricted (77–147 days) (Group A) or vice versa (Group B).

	Repeatability^#^
High repeatability (0.7 ≤ r ≤ 0.9)^1^	
3-Methyl-histidine	0.91
Alkaline phosphatase (ALP)	0.90
Betaine	0.78
Carnosine	0.78
1-Methyl-histidine	0.76
Indoleacetic acid*	0.76
Moderate repeatability (0.4 ≤ r < 0.7)^1^	
Homoarginine	0.69
δ^13^C	0.68
Creatinine	0.66
Alpha-aminoadipic acid	0.65
Aspartate transaminase (AST)	0.64
Lactic acid	0.63
Glutamate	0.61
Glucose	0.60
5-Aminovaleric acid	0.59
Tryptophane	0.58
β-Aminobutyric acid*	0.57
Alanine transaminase (ALT)	0.56
Symmetric dimethylarginine	0.55
Indolepropionic acid*	0.55
Anserine	0.49
IGF-1	0.48
δ^15^N	0.48
Arginine	0.47
Trans-4-hydroxyproline	0.47
Kynurenine	0.47
Valine	0.44
Tyrosine	0.43
Alanine	0.42
Asymmetric dimethylarginine	0.41

^#^Repeatability defined as the tendency of individuals to maintain their ranking over time based on the plasma concentration of metabolites and calculated as the intraclass correlation coefficient (see “Methods”).

^1^According to Martin and Bateson, 1986.

*Microbial origin.

The effect of RFI and feeding level on plasma parameters analysed from the 12 bulls identified as extreme RFI animals in group A and B are presented in Supplementary Tables 3 and 4. In group A animals, the plasma concentrations of three amino acid-related metabolites (alpha-aminoadipic acid, asymmetric dimethylarginine and carnosine) along with three amino acids (methionine, alanine and isoleucine) and one hormone (IGF-1) were lower (P < 0.05) in efficient compared to inefficient RFI counterparts (Table 6). This pattern remained consistent whether they were fed ad libitum (Test 1) or under restriction (Test 2), as evidenced by the absence of an RFI × feeding level interaction (P ≥ 0.39). These metabolites were not impacted by feeding level (P ≥ 0.07). Only the plasma concentration of alpha-aminoadipic acid reached significant differences after FDR corrections (FDR < 0.05). In group B animals, only the concentrations of three plasma metabolites (5-amino valeric acid, serine and betaine) were significantly and positively correlated with ad-libitum RFI values in both test periods (Table 7). These three metabolites were also impacted by feeding level (P < 0.001), but they were correlated with ad-libitum RFI values in the same way across both feeding levels (RFI × feeding level; P ≥ 0.11). Among them, only the plasma concentration of 5-aminovaleric acid was significantly correlated after FDR corrections (FDR < 0.05).

**Table 6:** Plasma metabolites and hormones affected by residual feed intake (RFI) in 12 growing-fattening bulls identified as extreme RFI animals during ad libitum feeding (0–70 days) and subsequently analysed during feed restriction (78–148 days) (Group A).

	Group A–Ad-libitum (0–70 days)		Group A—Restricted^1^ (78–148 days)		Fold change	SEM	*P value*		
	Low-RFI	High-RFI	Low-RFI	High-RFI			RFI	FL	RFI × FL
Number of animals	6	6	6	6					
Amino acid related, μM									
Alpha-Aminoadipic acid	1.13	1.83	0.983	1.48	1.57	0.142	0.003*	0.071	0.437
Asymmetric Dimethylarginine	0.933	1.10	0.933	1.03	1.14	0.050	0.023	0.520	0.520
Carnosine	18.0	23.7	16.8	22.2	1.32	1.85	0.042	0.249	0.881
Amino acids, μM									
Methionine	20.2	24.7	20.8	27.7	1.28	2.08	0.022	0.400	0.588
Alanine	197	230	176	218	1.20	14.5	0.049	0.198	0.718
Isoleucine	97.0	109	92.8	104	1.12	5.16	0.045	0.413	0.974
Hormones, ng/mL									
IGF-1	417	638	381	536	1.47	66.9	0.049	0.100	0.397

*P values with a star are also FDR < 0.05.

^1^Animals in the second test were the same as in the first test and kept the same RFI ranking (lowest vs highest) observed ad-libitum.

**Table 7:** Plasma metabolites correlated with residual feed intake (RFI) in 12 growing-fattening bulls identified as extreme RFI animals during feed restriction (0–70 days) and subsequently analysed during ad libitum feeding (78–148 days) (Group B).

	Pearson correlation coefficient		*P value*^*1*^		
	Group B—Restricted (0–70 days)^1^	Group B—Ad-libitum (78–148 days)	RFI	FL	RFI × FL
5-Aminovaleric acid	0.72	0.74	0.005*	< 0.001*	0.110
Serine	0.52	0.80	0.012	< 0.001*	0.725
Betaine	0.58	0.57	0.042	< 0.001*	0.272
Sarcosine	− 0.03	0.73	0.166	< 0.001*	0.012

*P values with a star are also FDR < 0.05.

^1^Animals in the first RFI test were the same as those in the second RFI test and maintained the same RFI values (ranging from − 0.98 to 0.61 kg/days) observed ad libitum.

## Discussion

To distinguish the impact of RFI from feeding level on systemic plasma parameters, we conducted a comparative analysis involving feeding the same animals under ad-libitum and restricted feeding conditions. Instead of using feed restriction as a percentage of a previously recorded ad-libitum intake^21^, we applied it as a percentage of the animals’ body weight (i.e., a proxy of maintenance requirements). This approach ensured a uniform nutrient intake per unit of body weight across all evaluated animals during the restriction phase, effectively eliminating differences in feed intake between RFI groups during this phase. In this regard, during ad libitum feeding, we observed approximately 8–10% variation in feed intake (% BW) among selected extreme RFI animals. In contrast, during feed restriction, we successfully minimized this variation to < 1%, which was statistically nonsignificant (Table 3) and much lower than the 12% difference observed during restriction in a previous study conducted in cattle^21^. Likewise, no differences in DM intake relative to body weight were reported when two chicken lines divergent in RFI were evaluated during feed restriction^20^. Similarly, studies in pigs^22^ and mink^23^ involving high and low RFI genetic lines or phenotypes, respectively, subjected to feed restriction were assessed at comparable feed intake levels. As argued by Metzler-Zebeli et al.^20^, maintaining a similar intake level between both RFI lines is essential for understanding the contributions of feeding behaviour (intake) versus host metabolic mechanisms in plasma metabolite concentrations. Consequently, to distinguish between the contributions of intake and host metabolic factors to variation in systemic plasma metabolite concentrations, we adopted a similar rationale in the present study.

**Figure 1:**
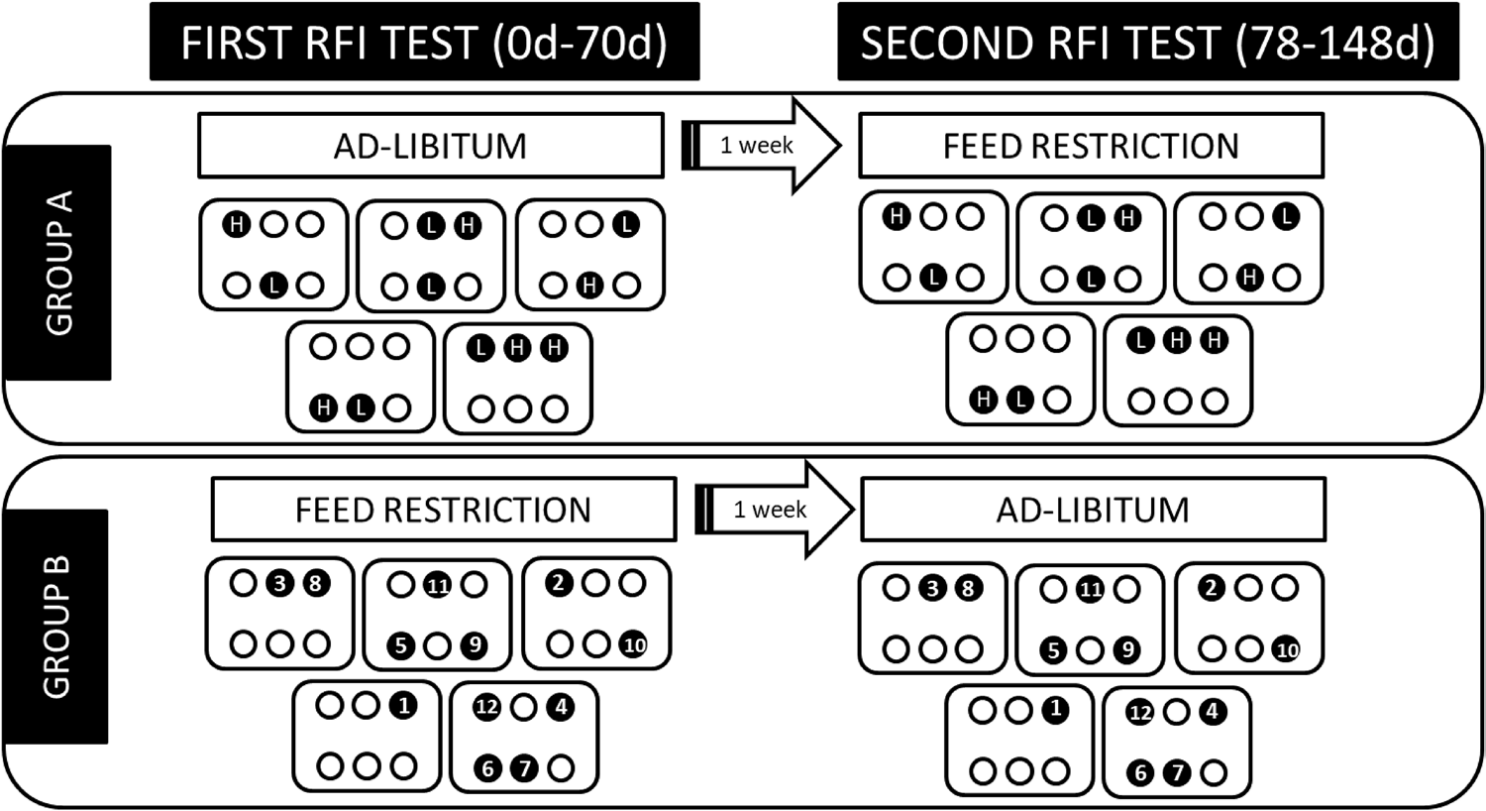
Experimental design of the study. Both Group A and Group B consisted of 30 growing-fattening beef cattle each (illustrated as circles within boxes, with boxes representing pens). During the first residual feed intake (RFI) test (0–70 days), Group A was fed ad libitum, while Group B underwent feed restriction. In both groups, 6 animals with the lowest and 6 with the highest RFI values were identified and sampled for blood (depicted as 24 black circles during the first RFI test). Following a 1-week transition period, the treatments were switched for the second RFI test (78–148 days): Group A underwent feed restriction, and Group B was fed ad libitum. Only the 24 cattle with the most extreme RFI values, sampled during the first RFI test, were resampled during the second RFI test (depicted as 24 black circles during the second RFI test). In Group A, the same RFI grouping established during ad libitum conditions (highest [H] and lowest [L] RFI) was maintained during feed restriction. In contrast, due to minimal RFI variation during feed restriction, the RFI ranking in group B (ranking number within black circles), as established during ad libitum conditions (second RFI test), was retained during feed restriction as well. For both groups and RFI tests, the retained RFI values were those obtained in ad-libitum conditions.

Conversely, our methodology for implementing feed restriction was responsible for the minimal variation observed in RFI values across individuals (coefficient of variation (CV) close to 0.6% during restriction vs. 4–5% during ad libitum feeding; Supplementary Table S2). Given that we ensured feed distribution was adjusted in accordance with BW during feed restriction, it is by design that nearly all the observed variation in DM intake was attributed to the metabolic BW predictor in the RFI model. Although we initially selected and sampled the most-extreme RFI animals during the first test period, extreme RFI bulls from Group B starting with feed restriction exhibited only a 1.7% CV in RFI, compared to an 11.3% CV in Group A during ad-libitum conditions (Supplementary Table S2). Consequently, and considering that RFI values were not correlated across feeding levels (Supplementary Table S2), we deemed the animals’ RFI phenotype to be that observed during ad-libitum conditions (first test for Group A and second test for Group B), maintaining the same ranking when animals underwent the restriction phase. As a result, and considering our experimental design (Fig. 1), relationships between RFI phenotype and plasma parameters were analysed by repeated measurement ANOVA in Group A (comprising two contrasted RFI groups selected during the first period [*ad-libitum*]) and by mixed model linear regression analysis in Group B (comprising animals with continuous RFI values observed during the second test [*ad-libitum*]).

### Plasma parameters associated with residual feed intake irrespective of the feeding level

Gaining a deeper understanding of the associations between blood plasma parameters and phenotypic traits is crucial before considering them as reliable biomarkers for aiding in genetic selection and precision feeding decisions. The findings of our study allow us, for the first time, to identify metabolites in beef cattle that show an association with the RFI phenotype even when animals are exposed to a uniform feeding level (i.e., eating the same amount of feed per kg of metabolic BW). This implies that these metabolites can be regarded as reliable RFI biomarkers, as they remain linked to this phenotype despite fluctuations in feed intake. The connection of these metabolites to RFI in a manner independent of intake would imply that they reflect host metabolic processes underlying individual variability in how animals convert feed and nutrients into growth^20^. This unique characteristic of a biomarker, combined with a proven genetic correlation, may confer a notable benefit when assisting multi-objective breeding programs aimed at enhancing metabolic feed efficiency regardless of intake. In this context, when the energy use efficiency was compared between divergent genetic RFI lines subjected to a similar feed intake (based on BW), it was concluded that selection for RFI in cattle may not necessarily lead to an improved efficiency of energy use^24^. They found that most of the metabolic differences observed across cattle RFI lines were attributed to variations in their feeding levels. Our results highlight the presence of some metabolic distinction between RFI groups that cannot be solely attributed to differences in feed intake levels.

It is important to note that the only metabolites associated with RFI independent of feed intake in our study (FDR < 0.05), namely, α-aminoadipic acid and 5-aminovaleric acid (also known as 5-aminopentoic acid), for Groups A and B, respectively, both belong to the same metabolic pathway: lysine degradation. Previous studies in beef cattle also found lower plasma concentrations of α-aminoadipic acid^25^ and 5-aminovaleric acid^17^ in efficient (low-RFI) compared to inefficient (high-RFI) cattle and proposed them as candidate biomarkers of RFI. One of the first studies to propose plasma metabolomics to identify RFI biomarkers in beef cattle^12^ also found that the lysine degradation pathway was linked to the RFI phenotype. The results from our team have similarly identified the lysine degradation pathway as a key metabolic pathway associated with differences in divergent RFI young bulls^11^. Furthermore, we have previously demonstrated that lysine plasma concentration, along with that of branched-chain amino acids, decreased in efficient compared to inefficient RFI heifers^15^. Likewise, studies in pigs have also linked plasma α-aminoadipic acid levels and the lysine degradation pathway to the RFI phenotype^26^.

Lysine degradation intermediates identified in this study may have both endogenous and microbial origins^27–29^. Consequently, our study does not allow us to distinguish whether these differences between efficient and inefficient RFI cattle originate from host metabolism or host-related microbiota. Interestingly, a recent study conducted in beef heifers^30^ identified lysine degradation as one of the unique pathways downregulated in the rumen of efficient RFI cattle. Similarly, in a study conducted in beef steers^31^, they found lower concentrations of several metabolites related to lysine metabolism, primarily N(6)-methylhistidine and cadaverine (the immediate precursor of 5-aminovaleric acid), in the rumen of more efficient RFI individuals. In a metatranscriptomic profiling study in beef cattle^32^, it was found that the lysine metabolism pathway, along with four other amino acids, was more active in the rumen microbiota of high-RFI individuals. Collectively, these results, combined with the findings of our study, may support the association between rumen microbiota composition or activity and the RFI phenotype^30,33^, which is not explained by differences in feed intake. However, further studies are warranted to assess whether these two RFI biomarkers, which remain consistent regardless of the feed intake level, are related to endogenous host or microbial metabolism.

Other amino acids and related metabolites were found to be associated (P < 0.05) with RFI, regardless of the feeding level; however, they did not reach statistical significance after FDR correction (FDR > 0.05), likely due to the limited number of animals used in this experiment (n = 6 per classification). Among these, it is interesting to note that isoleucine, one of the three BCAAs, was 12% higher in less efficient animals from Group A. The same trend was also observed for the other two BCAAs, leucine and valine, which tended to differ between RFI phenotypes, regardless of the feeding level (raw P values < 0.10; data not shown). This confirms results reported by others in beef cattle^11,14,16^ and growing lambs^34^, showing lower plasma concentrations of BCAAs in efficient RFI individuals. Our findings, even though they did not reach statistical significance after false positive correction, align with previous conclusions obtained in a meta-analysis of genome-wide association studies in beef cattle^18^, where BCAA degradation was identified as the only metabolic pathway associated with RFI. In contrast, the plasma concentration of none of the BCAAs was correlated with RFI values obtained in animals from Group B, those starting with feed restriction. Given the link between adiposity and BCAA plasma concentration in cattle^35^, we cannot rule out the possibility that slight differences in adipogenesis in animals from Group A vs. B (Table 1) may mask the relationships between BCAA and RFI in those animals starting with feed restriction (Group B). Finally, it is interesting to note that the plasma concentration of IGF-1, although not statistically significant after FDR correction, exhibited higher values in efficient RFI cattle (raw P value = 0.046), whether they were fed ad libitum or restricted to a similar feeding level as their inefficient counterparts (RFI × feeding level; P > 0.10). The plasma concentration of IGF-1 has been shown to correlate with RFI in various cattle studies^15,36^. However, diet-dependent relationships were highlighted in a review of the literature^4^, with a negative relationship, such as the one found in the present study, consistently observed only with low-concentrate and low-energy diets.

As opposed to lysine-degradation intermediates found in the present study, our results suggest that the plasma concentration of sarcosine, previously reported to differ across divergent RFI cattle fed diets rich in grass silage^37^, could be associated with this phenotype only because it covaries with feed intake (RFI × FL interaction; P = 0.012 in Group B; Table 7). Similar results were found for glycine, its natural precursor, in genetically divergent chicken lines for RFI when fed ad libitum or under restrictive feeding^20^. This finding is also consistent with previous studies, indicating that glycine is an amino acid whose plasma concentration in ruminants is inversely correlated with dietary protein intake^38^.

### The impact of feeding level on plasma parameters

Many plasma parameters evaluated here were significantly impacted by feeding level (FDR < 0.05), regardless of whether feed restriction was applied in the first or second test. The high correlation between feed intake and RFI, on the one hand, and feed intake and plasma metabolite concentration, on the other, may pose a challenge in determining whether plasma RFI biomarkers will remain consistent when animals are fed at similar feed intake levels. Among the plasma parameters which increased their values during feed restriction were some endogenous metabolites, such as methyl-histidines, which are known to increase during muscle degradation and mobilization in cattle^39^, as well as other specific markers of (1) lipid mobilization, such as NEFA (highest-fold change in our study) or individual fatty acids^40^ and (2) protein mobilization, such as δ^15^N values^41,42^. The values of these plasma markers of body mobilization agree with the effect of feeding level on the measured fat and muscle depth change, where feed restriction was associated with much lower tissue depth changes (close to 0 during the second test) compared to ad libitum feeding.

Furthermore, our results agree with previous findings showing that glycine and betaine plasma concentrations strongly increased when feed (protein) intake decreased in humans and mammals^43^ and during feed restriction in female mink^23^, respectively. In addition, we observed that serine and sarcosine, two metabolites related to glycine metabolism^43^, also increased their plasma concentration during feed restriction, which suggests that the serine-glycine biosynthesis pathway is strongly regulated by feed intake. On the other hand, few plasma parameters decreased their values during feed restriction (FDR < 0.05). The lower plasma concentrations of β-hydroxybutyrate and hippuric acid during feed restriction likely indicated lower absorption of rumen butyrate (one of the three main volatile fatty acids) and benzoic acids (aromatic compounds found in forages), their main substrates, respectively^44,45^. The third plasma metabolite that decreased in concentration when feed intake decreased was β-alanine. This result could be expected given that β-alanine is a breakdown product from the muscle-rich dipeptides anserine and carnosine, the latter also decreasing its concentration during feed restriction (raw P value = 0.018).

Our study revealed several plasma parameters that, although affected by feeding level, enabled the ranking of animals across time (between-feeding levels). The repeatability quantifies the proportion of total phenotypic variation that is attributed to differences between individuals and may suggest that genetic factors are likely to be important. The moderate-to-high repeatability observed for some plasma parameters in this study indicates that they are somehow inherent to animal metabolism beyond the effects of feeding level. To have confidence in their predictive ability, it is important that biomarkers consistently rank individual animals even when the environment changes. 5-Aminovaleric acid and α-aminoadipic acid, the only two metabolites related to RFI in a feeding level-independent manner, showed moderate (0.59) to high repeatability (0.65), respectively. Among the plasma parameters showing the highest repeatability between-feeding levels, there were endogenous metabolites such as 1 and 3-methylhistidine, alkaline phosphatase and carnosine and others with a known microbial origin, such as indoleacetic acid (major product of tryptophan metabolism by microbial population in the rumen^46^). Plasma metabolites such as methylhistidines and carnosine, with high repeatability across feeding levels, are endogenous metabolites that share a common feature: originating from skeletal muscle turnover and metabolism^47^. Furthermore, alkaline phosphatase is an enzyme related to bone turnover and metabolism, and its blood levels have been shown to be genetically regulated in mice, with a heritability of 0.56, and related to bone mass density^48^. Given that the proportion of carcass, comprising both muscle and bones, to total body weight are heritable traits in beef cattle^49^ (h^2^ = 0.51) it is not surprising that plasma metabolites reflecting muscle and bone turnover rates (mainly 3-methylhistidines and phosphatase alkaline) showed very high repeatability across time in the present study.

The plasma creatinine concentration showed moderate repeatability in our study (0.66), consistent with findings in dairy cows^50,51^. Most highly conserved metabolites are also highly heritable^52^ and less influenced by environmental factors. Indeed, in a genome-wide association study conducted in crossbred beef cattle, creatinine ranked as the second highest plasma metabolite in terms of heritability^53^ (h^2^ = 0.35) and showed moderate heritability (0.45) when measured in cow’s milk^50^. Plasma creatinine is a product of muscle metabolism and has been proposed to reflect lean body mass in ruminants^54^, an animal trait with moderate heritability and phenotypic and genetic correlations with RFI^49^. Although several studies identified a relationship between plasma creatinine concentration and RFI^11,12^, overall, we observed no correlation in our study. The study by Karisa et al.^12^ highlighted that, in addition to explaining 26% of the phenotypic RFI variation observed in beef cattle, plasma creatinine also contributed to explaining 13% of DM intake variation. Further studies are needed to confirm whether the relationship between plasma creatinine concentration and RFI is confounded by covariations in DM intake.

## Conclusions

In summary, our study revealed that metabolites belonging to the lysine degradation pathway, including α-aminoadipic acid and 5-aminovaleric acid, emerged as consistent and repeatable biomarkers for RFI. These metabolites displayed a remarkable characteristic of remaining linked to RFI regardless of fluctuations in feed intake, likely reflecting underlying host metabolic processes associated with individual variability in feed efficiency. Our results support the notion that some of the phenotypic variance of RFI may not be solely attributed to differences in feed intake. Under the conditions of this study, we cannot confirm our hypothesis that the association between plasma branched-chain amino acids and RFI is independent of fluctuations in feeding level. Furthermore, our research has underscored changes in plasma parameter values during feed restriction, primarily associated with muscle and adipose tissue mobilization. Further investigations are needed to further validate our findings and to determine whether the identified RFI biomarkers have their origins in microbial or host metabolism. This step is essential before considering their utilization in assisting breeding programs and precision feeding strategies.

## Methods

This study was conducted at the Teagasc Animal & Grassland, Research and Innovation Centre (Grange, Ireland) between 2021 and 2022. All experimental procedures involving animals were approved by the Teagasc Animal Ethics Committee (TAEC No. TAEC2021-304) and the Irish Health Products Regulatory Authority (HPRA, License number AE19132/P141) in accordance with the European Union (Protection of Animals used for Scientific Purposes) Regulations 2012 (S.I. No. 543/2012). Prior to commencing the study, all animals were treated for the control of internal parasites, skin lice, and vaccinated against clostridial and respiratory diseases. This experiment complied with the ARRIVE guidelines.

### Animals, dietary treatments and experimental design

Sixty Charolais crossbred young bulls, with an average age of 13 ± 1.4 months and an initial body weight (BW) of 427 ± 20.4 kg, were utilized in a crossover design study, encompassing two 70-d FE test periods, as illustrated in Fig. 1. Bulls were allocated to one of two ‘groups’ (Group A, n = 30, and Group B, n = 30) in a randomized block design, with blocking based on initial live weight. Bulls were accommodated in a slatted floor building, with five pens of six animals per treatment. Pens within treatment were distributed throughout the building.

Prior to the commencement of the first RFI test, animals underwent a 21-day adaptation period to acclimatize to the diet and environment. Group A was provided ad libitum feeding during the initial 70-day RFI test, followed by a period of feed restriction in the subsequent 70-day RFI test. Conversely, Group B experienced the opposite sequence, commencing with feed restriction in the first test and then transitioning to ad libitum feeding for the second test. A one-week transition interval was implemented between the two RFI test periods.

Throughout the study, all animals were fed the same total mixed ration (TMR), which comprised a blend of grass silage and concentrate at a 50:50 ratio on a DM basis. The concentrate portion of the diet was formulated using rolled barley, soybean meal, and cane molasses plus minerals and vitamins. The TMR had a DM concentration of 528 g/kg, a crude protein (CP) content of 138 g/kg DM, and an estimated net energy (NE) concentration for maintenance and growth of 1.58 Mcal/kg DM^2^. The chemical composition and in vitro digestibility of the grass silage and concentrate feed offered are detailed in Supplementary Table S1. Animals had free access to clean, fresh drinking water.

Bulls were individually offered their respective dietary feeding level through electronically controlled Calan doors (American Calan Inc., Northwood, NH, USA) once daily after 08:00. Ad libitum feed intake was based on a targeted refusal rate of approximately 5–10%; refused feed was weighed daily and discarded twice weekly. Feed restriction was set at an intermediate point between ad-libitum conditions and a feeding level that only met the energy requirements for maintenance (zero-growth). Considering the theoretical chemical composition and dietary values of the feed, along with the observed feeding level during the adaptation period (1.88% of body weight), the feed restriction level equated to 1.45% of body weight on a DM basis for each individual animal. Accordingly, the quantity of feed offered to individual animals in the restricted group(s) was adjusted every 2 weeks based on individual animal live weight. This adjustment resulted in a projected average daily live weight gain of 0.62 kg for a 400 kg young bull, as opposed to the 1.35 kg/day expected under ad-libitum conditions.

Representative samples of the grass silage and concentrates offered were collected three and two times weekly, respectively. Silage DM was determined by oven drying samples at 85 °C for 16 h; DM values were corrected for loss of volatiles using equation 1 proposed in^55^. Concentrate samples were oven-dried at 98 °C for 16 h to determine DM. Concentrations of crude protein, neutral detergent fibre, acid detergent fibre, ash, starch and oil-B and in vitro measures of digestibility were determined in the feedstuffs (wet chemistry), as described previously^56^.

Animal live weight was recorded on two consecutive days at the beginning and the end of each feed efficiency test period and weekly throughout using a calibrated scale. Weighing was carried out in the morning prior to feeding. The depth of subcutaneous fat (13th rib, third lumbar vertebra and the rump) and *M. longissimus* muscle (third lumbar vertebra) was measured at the beginning and end of each test period using an automatic real-time scanner (model—ECM ExaGo Veterinary scanner, with a 3.5 MHz linear transducer, IMV imaging, Meath, Ireland), as described previously^57^.

### Blood sampling and analysis

Blood was collected prior to the morning feeding at the end of the first FE test period from the 12 most divergent RFI (6 high-RFI and 6 low-RFI) animals from each group; the same animals were blood sampled again at the end of the second FE test period (Fig. 1). Blood samples were obtained using jugular venipuncture into two 9-mL evacuated vials containing lithium heparin (Vacuette; Cruinn Diagnostics, Dublin, Ireland) and two 8.5-mL evacuated vials containing silica clot activator (BD Vacutainer SST (II) Advance, Unitech). The lithium heparin blood tubes were placed in ice, and the serum separator tubes were left for approximately 24 h at 4 °C to permit clotting prior to centrifugation (1600 × g for 10 min). The resulting plasma and serum were divided into 1.5 mL plastic vials and stored at − 80 °C until subsequent analyses. The resulting supernatant was subjected to metabolomic profiling analysis at the core lab of Biocrates Life Science AG (Innsbruck, Austria).

All 48 plasma samples were stored upon receipt at − 80 °C and thawed on ice before analysis. The thawed samples were immediately pipetted to ensure that all samples were analyzed within 30 min, thereby avoiding any changes that could affect metabolite levels. To directly quantify known metabolites, we employed a commercially available kit (MxP Quant 500 Assay) utilizing liquid chromatography-tandem mass spectrometry, allowing for the quantification of up to 106 metabolites. This targeted metabolic approach included the analysis of one alkaloid, one amine oxide, 20 amino acids, 30 amino acid-related metabolites, 14 bile acids, nine biogenic amines, one carbohydrate and related, seven carboxylic acids, one cresol, 12 fatty acids, four hormones and related, four indoles and derivatives, two nucleobases and related and one vitamin. Only 71 out of the 106 targeted metabolites had values above the limit of detection for all observations and were used in further analysis. Among these metabolites, several were previously identified as candidate RFI biomarkers through untargeted and targeted approaches in beef cattle studies^11,12,15^. Additionally, the plasma samples were subjected to spectrophotometric quantification of various metabolites, including glucose, urea, nonesterified fatty acids, β-hydroxybutyrate, as detailed in Jorge-Smeding et al.^11^ and hepatic transaminases as previously described^58^, some of which were identified as potential RFI biomarkers by Richardon et al.^59^. The natural abundance of ^15^N, a promising biomarker of FE in beef cattle^60^, was determined following the methodology outlined in Cantalapiedra-Hijar et al.^61^. Finally, using commercial ELISA kits, plasma insulin (bovine insulin ELISA kit Cat. no. EB0092, FineTest, China) and IGF-1 (bovine IGF-1 ELISA kit Cat. No. 221121, Mediadiagnost, GMbH, Tübingen, Germany) concentrations were also analysed from blood serum samples. Both hormones were also found to differ across extreme RFI cattle in previous studies^11,15,62^.

### Calculations and statistical analysis

The RFI values of each animal were computed as the difference between observed and predicted DM intake. DM intake was predicted from the following factors: block, pen within block, mid-test metabolic body weight (mBW^0.75^), ADG and changes in backfat and muscle ultrasound measurements between the end and beginning of the test as commonly recommended^5^. This computation followed the equation provided below:

$${\text{DM}}\;{\text{ intake}}\, = \,\beta 0\, + \,{\text{Block}}\, + \,{\text{Pen}}\, + \,\beta {1}\, \times \,{\text{mBW}}^{{0.{75}}} \, + \,\beta {2}\, \times \,{\text{ADG}}\, + \,\beta {3}\, \times \,{\text{ultrasound }}\;{\text{depth }}\;{\text{change}}\, + \,\varepsilon ;$$

where β0 represents the regression intercept, β1 the partial regression coefficient of mean metabolic BW, β2 the partial regression coefficient of ADG, β3 the partial regression for the ultrasound depth change and ε is the residual error of the regression for each animal, or RFI.

The ADG used in the RFI model was determined as the regression of BW over time, except in the case of ADG measured in group B during the first test period, where nonlinear growth (r2 < 0.9 for some animals) necessitated calculation as the difference between final BW and initial BW divided by 70 days.

Separate RFI models were established for each group and test period, totaling four conditions. Consequently, the models featured distinct significant variables depending on the specific condition, as outlined in Supplementary Table S2. All statistical analyses were performed using R software (version 2021.09.0; R Core Team, 2021). Variables that did not adhere to a normal distribution (P < 0.05 after the Shapiro‒Wilk test) were log-transformed prior to analysis. We utilized a mixed-effects model analysis with the nlme package in R, consistently including “animal” as a random effect when repeated measures were analysed. Different fixed effects were examined in separate analyses. To assess the effect of feeding level, we included it as a fixed effect, along with the effect of the test period and their interaction. To evaluate the effect of RFI across the two feeding levels, both factors and their interaction were included as fixed effects in the model. However, as elaborated later, we treated the RFI effect differently according to the Group: as a qualitative variable in Group A (categorizing animals into high vs. low RFI extremes) and as a continuous variable in Group B (with values ranging from positive to negative, including values close to zero). The raw P values obtained from the analysis of plasma variables (n = 83) were adjusted using the Benjamini‒Hochberg false discovery rate (FDR) correction. The significance threshold was set at FDR ≤ 0.05. However, because some variables have already been identified as candidate plasma biomarkers in previous studies, we also examined raw P values. In this context, the likelihood of a false positive is lower when compared to new plasma biomarkers. The repeatability of plasma parameters across the two feeding levels was calculated as the intraclass correlation coefficient (i.e., animal variance divided by the sum of animal variance and residual variance) in a model that explained the variation in plasma parameters using feeding level, test period, and their interaction as fixed effects, along with the random animal effect.

### Supplementary Information

Supplementary Tables.

## Data Availability

Data is provided within the manuscript or supplementary information files. Raw data generated and/or analysed during the current study are available from the corresponding author upon reasonable request.
