# Supplementary material for: Some plasma biomarkers of residual feed intake in beef cattle remain consistent regardless of intake level
Source: Sci Rep. 2024 Apr 12;14:8540. doi: 10.1038/s41598-024-59253-7 (PMC11014993; doi:10.1038/s41598-024-59253-7)
Supplement: Supplementary file 1 — Supplementary Tables. [file 41598_2024_59253_MOESM1_ESM.docx]

|  | | |
| --- | --- | --- |
|  | Grass silage | Concentrate |
| Dry matter (g/kg) | 237 (8.1) | 818 (10.1) |
| Crude protein (g/kg DM) | 129 (4.6) | 147 (11.0) |
| Neutral detergent fibre (g/kg DM) | 534 (13.3) | 176 (20.6) |
| Acid detergent fibre (g/kg DM) | 355 (11.6) | 63 (12.1) |
| Ash (g/kg DM) | 97 (3.0) | 55 (3.4) |
| Starch (g/kg DM) | - | 504 (18.1) |
| Oil-B (g/kg DM) | - | 27 (2.0) |
| Dry matter digestibility (g/kg) | 737 (10.5) | - |
| Organic matter digestibility (g/kg) | 728 (11.9) | - |
| Digestible organic matter in the dry matter (g/kg DM) | 657 (11.5) | - |
| Neutral cellulase gammanase digestibility (g/kg DM) | - | 864 (3.7) |

**Supplementary Table 1.** Dry matter, chemical composition and *in vitro* digestibility (s.d.) of the grass silage and the concentrate feed offered in the Total Mixed Ration (TMR)

**Supplementary Table 2.** Residual feed intake (RFI) models, between-animal variability in RFI and repeatability of RFI values from growing-fattening bulls fed ad libitum and then restricted (Group A) or vice versa (Group B) during two consecutive 70-d feed efficiency tests

|  |  | Group A | |  | Group B | |
| --- | --- | --- | --- | --- | --- | --- |
|  |  | Ad-libitum  (Test 1; 0-70d) | Restricted  (Test 2; 78-148d) |  | Restricted  (Test 1; 0-70d) | Ad-libitum  (Test 2; 78-148d) |
| Number of animals |  | 29 | 28 |  | 30 | 30 |
| Tested variables in the RFI model and significance^1^ |  |  |  |  |  |  |
| Block |  | ** | *** |  | *** | *NS* |
| Pen |  | P <0.10 (not included) | *** |  | * | *NS* |
| Mean metabolic body weight |  | *** | *** |  | *** | *** |
| Average daily gain |  | ** | *NS* |  | *NS* | ** |
| Muscle depth change |  | *NS* | * |  | *NS* | *NS* |
| Rump fat depth change |  | P <0.10 (not included) | *NS* |  | *NS* | *NS* |
| Lumbar fat depth change |  | *NS* | *NS* |  | *NS* | *NS* |
| Rib fat depth change |  | *NS* | *NS* |  | *NS* | *NS* |
| Coefficient of determination (R^2^) from the retained RFI model |  | 0.81 | 0.98 |  | 0.99 | 0.51 |
| Coefficient of variation of RFI^2^, % |  | 3.85 | 0.59 |  | 0.64 | 5.08 |
| Correlation of RFI values across feeding levels (within-group) |  |  |  |  |  |  |
| Pearson coefficient |  | 0.34 (P = 0.08) | |  | -0.09 (P = 0.68) | |
| Spearman coefficient |  | 0.33 (P = 0.09) | |  | -0.004 (P = 0.95) | |

^1^ *** P<0.001; ** P<0.01; *P <0.05; NS: P>0.10

^2^ Calculated as the standard deviation of residual feed intake divided by the mean DM intake

**Supplementary Table 3**. The effect of residual feed intake (RFI) phenotype and feeding level (FL) on plasma parameters from 12 growing-fattening bulls identified as extreme RFI animals during ad libitum feeding (0 d – 70 d) and subsequently analyzed during feed restriction (78 d – 148 d) (Group A).

|  |  | Group A – Ad-libitum (0-70d) | | |  | Group A – Restricted^1^ (78-148d) | | |  | Fold |  |  |  | *P value* | | |
| --- | --- | --- | --- | --- | --- | --- | --- | --- | --- | --- | --- | --- | --- | --- | --- | --- |
| Plasma parameter |  | Low-RFI |  | High-RFI |  | Low-RFI |  | High-RFI |  | change |  | SEM |  | RFI | FL | RFI × FL |
| Trimethylamine N-oxide |  | 7.14 |  | 13.2 |  | 16.0 |  | 17.9 |  | 1.34 |  | 7.97 |  | 0.63 | 0.42 | 0.80 |
| 1-Methylhistidine |  | 6.26 |  | 6.80 |  | 8.09 |  | 8.67 |  | 1.08 |  | 0.662 |  | 0.53 | <0.001* | 0.95 |
| 3-Methylhistidine |  | 4.72 |  | 6.08 |  | 5.75 |  | 6.60 |  | 1.21 |  | 0.832 |  | 0.36 | 0.002* | 0.22 |
| 5-Aminovaleric acid |  | 1.35 |  | 1.03 |  | 1.34 |  | 1.47 |  | 0.92 |  | 0.227 |  | 0.42 | 0.25 | 0.63 |
| Alpha-amino-butyric acid |  | 10.0 |  | 15.0 |  | 8.82 |  | 10.9 |  | 1.38 |  | 1.31 |  | 0.05 | 0.01 | 0.32 |
| Asymmetric dimethylarginine |  | 0.933 |  | 1.10 |  | 0.933 |  | 1.03 |  | 1.14 |  | 0.050 |  | 0.02 | 0.52 | 0.52 |
| Alpha-Aminoadipic acid |  | 1.13 |  | 1.83 |  | 0.983 |  | 1.48 |  | 1.57 |  | 0.142 |  | 0.003* | 0.07 | 0.44 |
| L-anserine |  | 0.358 |  | 0.383 |  | 0.357 |  | 0.450 |  | 1.17 |  | 0.036 |  | 0.20 | 0.26 | 0.25 |
| Betaine |  | 124 |  | 110 |  | 124 |  | 112 |  | 0.90 |  | 9.08 |  | 0.28 | 0.87 | 0.92 |
| Carnosine |  | 18.0 |  | 23.7 |  | 16.8 |  | 22.2 |  | 1.32 |  | 1.85 |  | 0.04 | 0.25 | 0.88 |
| Citrulline |  | 69.5 |  | 67.7 |  | 81.4 |  | 69.4 |  | 0.91 |  | 5.35 |  | 0.45 | 0.11 | 0.10 |
| Creatinine |  | 134 |  | 144 |  | 138 |  | 149 |  | 1.08 |  | 10.7 |  | 0.44 | 0.61 | 0.97 |
| Cystine |  | 8.82 |  | 10.0 |  | 11.7 |  | 14.5 |  | 1.19 |  | 1.33 |  | 0.19 | 0.01 | 0.52 |
| Homoarginine |  | 3.76 |  | 3.70 |  | 2.88 |  | 2.65 |  | 0.96 |  | 0.411 |  | 0.78 | 0.006 | 0.77 |
| Homocysteine |  | 6.36 |  | 6.60 |  | 7.09 |  | 9.04 |  | 1.16 |  | 1.49 |  | 0.48 | 0.31 | 0.58 |
| Kynurenine |  | 5.95 |  | 7.70 |  | 5.03 |  | 6.13 |  | 1.26 |  | 0.653 |  | 0.11 | 0.02 | 0.49 |
| Methionine-sulfoxide |  | 0.353 |  | 0.389 |  | 0.333 |  | 0.391 |  | 1.14 |  | 0.0327 |  | 0.18 | 0.78 | 0.74 |
| Ornithine |  | 52.9 |  | 55.4 |  | 57.1 |  | 62.5 |  | 1.07 |  | 4.11 |  | 0.41 | 0.15 | 0.70 |
| Sarcosine |  | 1.91 |  | 2.07 |  | 1.78 |  | 2.01 |  | 1.11 |  | 0.267 |  | 0.55 | 0.67 | 0.87 |
| Symmetric dimethylarginine |  | 0.528 |  | 0.618 |  | 0.534 |  | 0.575 |  | 1.12 |  | 0.0386 |  | 0.19 | 0.54 | 0.43 |
| Trans-4-hydroxyproline |  | 29.8 |  | 33.8 |  | 31.7 |  | 33.1 |  | 1.09 |  | 2.69 |  | 0.41 | 0.78 | 0.53 |
| Alanine |  | 197 |  | 230 |  | 176 |  | 218 |  | 1.20 |  | 14.5 |  | 0.05 | 0.20 | 0.72 |
| Arginine |  | 92.6 |  | 96.8 |  | 105 |  | 101 |  | 1.00 |  | 7.83 |  | 0.65 | 0.26 | 0.99 |
| Asparagine |  | 31.4 |  | 34.0 |  | 26.9 |  | 31.6 |  | 1.13 |  | 2.47 |  | 0.17 | 0.19 | 0.66 |
| Aspartic acid |  | 8.81 |  | 11.1 |  | 6.89 |  | 6.88 |  | 1.15 |  | 1.38 |  | 0.47 | 0.03 | 0.35 |
| Cysteine |  | 21.7 |  | 24.8 |  | 27.3 |  | 35.4 |  | 1.23 |  | 2.56 |  | 0.07 | 0.005 | 0.31 |
| Glutamine |  | 403 |  | 409 |  | 375 |  | 402 |  | 1.04 |  | 22.1 |  | 0.47 | 0.46 | 0.64 |
| Glutamic acid |  | 73.7 |  | 103 |  | 65.9 |  | 75.7 |  | 1.28 |  | 10.1 |  | 0.15 | 0.02 | 0.17 |
| Glycine |  | 411 |  | 407 |  | 521 |  | 443 |  | 0.91 |  | 32.4 |  | 0.28 | 0.05 | 0.23 |
| Histidine |  | 49.4 |  | 56.7 |  | 59.5 |  | 63.1 |  | 1.10 |  | 6.13 |  | 0.46 | 0.13 | 0.71 |
| Isoleucine |  | 97.0 |  | 109 |  | 92.8 |  | 104 |  | 1.12 |  | 5.16 |  | 0.05 | 0.41 | 0.97 |
| Leucine |  | 88.6 |  | 104 |  | 91.9 |  | 103 |  | 1.15 |  | 6.72 |  | 0.10 | 0.85 | 0.75 |
| Lysine |  | 61.4 |  | 70.2 |  | 62.6 |  | 72.2 |  | 1.15 |  | 6.27 |  | 0.20 | 0.78 | 0.95 |
| Methionine |  | 20.2 |  | 24.7 |  | 20.8 |  | 27.4 |  | 1.28 |  | 2.08 |  | 0.02 | 0.40 | 0.59 |
| Phenylalanine |  | 47.0 |  | 51.8 |  | 46.8 |  | 48.0 |  | 1.06 |  | 3.47 |  | 0.49 | 0.46 | 0.51 |
| Proline |  | 63.7 |  | 68.4 |  | 63.6 |  | 73.0 |  | 1.11 |  | 4.23 |  | 0.13 | 0.60 | 0.59 |
| Serine |  | 101 |  | 82.9 |  | 96.3 |  | 100 |  | 0.93 |  | 11.0 |  | 0.54 | 0.56 | 0.34 |
| Threonine |  | 45.7 |  | 45.6 |  | 46.9 |  | 56.5 |  | 1.10 |  | 5.17 |  | 0.38 | 0.27 | 0.37 |
| Tryptophan |  | 38.4 |  | 48.5 |  | 38.3 |  | 45.8 |  | 1.23 |  | 3.59 |  | 0.07 | 0.60 | 0.62 |
| Tyrosine |  | 46.1 |  | 57.8 |  | 45.8 |  | 51.7 |  | 1.19 |  | 4.33 |  | 0.10 | 0.42 | 0.47 |
| Valine |  | 163 |  | 190 |  | 168 |  | 180 |  | 1.12 |  | 10.1 |  | 0.08 | 0.97 | 0.59 |
| Cholic acid |  | 6.25 |  | 8.36 |  | 9.53 |  | 15.9 |  | 1.54 |  | 3.14 |  | 0.21 | 0.11 | 0.51 |
| Chenodeoxycholic acid |  | 0.339 |  | 0.433 |  | 0.481 |  | 0.632 |  | 1.30 |  | 0.168 |  | 0.48 | 0.33 | 0.86 |
| Deoxycholic acid |  | 0.66 |  | 1.08 |  | 1.77 |  | 2.41 |  | 1.44 |  | 0.729 |  | 0.49 | 0.13 | 0.88 |
| Glycocholic acid |  | 1.88 |  | 2.69 |  | 2.07 |  | 4.61 |  | 1.85 |  | 0.922 |  | 0.17 | 0.12 | 0.20 |
| Glycochenodeoxycholic acid |  | 0.421 |  | 0.510 |  | 0.425 |  | 0.718 |  | 1.45 |  | 0.139 |  | 0.23 | 0.41 | 0.43 |
| Glycodeoxycholic acid |  | 0.925 |  | 1.26 |  | 1.36 |  | 2.04 |  | 1.44 |  | 0.388 |  | 0.26 | 0.11 | 0.63 |
| Glycolithocholic acid |  | 0.068 |  | 0.077 |  | 0.070 |  | 0.096 |  | 1.25 |  | 0.0164 |  | 0.30 | 0.55 | 0.60 |
| Taurocholic acid |  | 0.130 |  | 0.219 |  | 0.164 |  | 0.488 |  | 2.40 |  | 0.114 |  | 0.13 | 0.17 | 0.28 |
| Taurochenodeoxycholic acid |  | 0.070 |  | 0.091 |  | 0.068 |  | 0.181 |  | 1.97 |  | 0.0432 |  | 0.17 | 0.32 | 0.30 |
| Taurolithocholic acid |  | 0.153 |  | 0.233 |  | 0.192 |  | 0.440 |  | 1.95 |  | 0.113 |  | 0.21 | 0.26 | 0.43 |
| Beta-alanine |  | 3.75 |  | 4.14 |  | 3.14 |  | 2.86 |  | 1.02 |  | 0.573 |  | 0.93 | 0.12 | 0.56 |
| Gamma-aminobutyric acid |  | 0.108 |  | 0.125 |  | 0.0897 |  | 0.110 |  | 1.19 |  | 0.0109 |  | 0.17 | 0.10 | 0.87 |
| Putrescine |  | 0.115 |  | 0.109 |  | 0.101 |  | 0.123 |  | 1.07 |  | 0.0211 |  | 0.75 | 0.98 | 0.42 |
| Serotonin |  | 1.56 |  | 4.36 |  | 1.11 |  | 1.56 |  | 2.22 |  | 1.82 |  | 0.39 | 0.38 | 0.52 |
| Dodecanedioic acid |  | 0.269 |  | 0.271 |  | 0.313 |  | 0.314 |  | 1.01 |  | 0.038 |  | 0.97 | 0.22 | 0.98 |
| Hippuric acid |  | 75.9 |  | 74.9 |  | 60.6 |  | 58.1 |  | 0.97 |  | 8.58 |  | 0.85 | 0.09 | 0.93 |
| Lactic acid |  | 4214 |  | 3110 |  | 3906 |  | 3939 |  | 0.87 |  | 1012 |  | 0.70 | 0.62 | 0.28 |
| p-cresol sulfate |  | 54.7 |  | 62.9 |  | 53.5 |  | 64.6 |  | 1.18 |  | 5.36 |  | 0.20 | 0.94 | 0.65 |
| Arachidonic acid |  | 0.855 |  | 1.39 |  | 0.927 |  | 1.19 |  | 1.45 |  | 0.279 |  | 0.25 | 0.78 | 0.56 |
| Docosahexaenoic acid |  | 0.770 |  | 0.924 |  | 0.823 |  | 0.982 |  | 1.20 |  | 0.101 |  | 0.15 | 0.59 | 0.98 |
| Eicosapentanoic acid |  | 0.223 |  | 0.279 |  | 0.229 |  | 0.295 |  | 1.27 |  | 0.0343 |  | 0.12 | 0.74 | 0.89 |
| Octadecenoic acid |  | 38.9 |  | 49.2 |  | 82.0 |  | 103 |  | 1.26 |  | 14.2 |  | 0.38 | 0.009 | 0.73 |
| Octadecadienoate |  | 4.14 |  | 5.54 |  | 8.53 |  | 11.2 |  | 1.32 |  | 1.54 |  | 0.24 | 0.007 | 0.69 |
| Indoleacetic acid |  | 0.597 |  | 0.656 |  | 0.609 |  | 0.624 |  | 1.06 |  | 0.0947 |  | 0.77 | 0.84 | 0.64 |
| Indolepropionic acid |  | 3.58 |  | 4.94 |  | 1.57 |  | 2.48 |  | 1.44 |  | 0.772 |  | 0.28 | <0.001* | 0.63 |
| Indoxyl sulfate |  | 1.80 |  | 2.70 |  | 2.03 |  | 2.19 |  | 1.28 |  | 0.271 |  | 0.09 | 0.60 | 0.19 |
| Choline |  | 12.3 |  | 16.3 |  | 12.1 |  | 13.1 |  | 1.20 |  | 1.93 |  | 0.22 | 0.41 | 0.46 |
| Glucose |  | 0.758 |  | 0.830 |  | 0.844 |  | 0.893 |  | 1.08 |  | 0.0656 |  | 0.50 | 0.06 | 0.75 |
| Non-esterified fatty acids |  | 0.078 |  | 0.100 |  | 0.238 |  | 0.295 |  | 1.25 |  | 0.0473 |  | 0.42 | 0.004* | 0.72 |
| Beta-hydroxyburytrate |  | 0.355 |  | 0.424 |  | 0.300 |  | 0.316 |  | 1.13 |  | 0.0283 |  | 0.16 | 0.02 | 0.38 |
| Urea |  | 0.119 |  | 0.133 |  | 0.139 |  | 0.153 |  | 1.11 |  | 0.0126 |  | 0.40 | 0.05 | 0.99 |
| Alanine aminotransferase |  | 17.7 |  | 18.8 |  | 21.7 |  | 21.5 |  | 1.02 |  | 1.71 |  | 0.86 | 0.02 | 0.60 |
| Aspartate aminotransferase |  | 64.2 |  | 61.3 |  | 75.3 |  | 68.5 |  | 0.93 |  | 4.55 |  | 0.40 | 0.02 | 0.58 |
| Alkaline phosphatase |  | 209 |  | 189 |  | 256 |  | 238 |  | 0.92 |  | 44.9 |  | 0.76 | 0.002* | 0.91 |
| Gamma-glutamyl transferase |  | 18.5 |  | 14.9 |  | 21.0 |  | 22.1 |  | 0.94 |  | 2.20 |  | 0.58 | 0.04 | 0.28 |
| Natural abundance of ^15^N |  | 5.60 |  | 5.68 |  | 5.53 |  | 5.68 |  | 1.02 |  | 0.133 |  | 0.50 | 0.72 | 0.67 |
| Insulin |  | 460 |  | 189 |  | 230 |  | 162 |  | 0.51 |  | 115 |  | 0.21 | 0.23 | 0.33 |
| IGF-1 |  | 417 |  | 638 |  | 381 |  | 536 |  | 1.47 |  | 66.9 |  | 0.05 | 0.10 | 0.40 |

* P values with a star are also FDR <0.05

^1^Animals in the second test were the same as in the first test and kept the same RFI ranking (lowest vs highest) observed ad-libitum

**Supplementary Table 4.** Correlation between plasma parameters and residual feed intake (RFI) in 12 growing-fattening bulls identified as extreme RFI animals during feed restriction (0 d-70 d) and subsequently sampled during ad libitum feeding (78-148 d).

|  |  | Pearson correlation coefficient | | |  | *P value^1^* | | |
| --- | --- | --- | --- | --- | --- | --- | --- | --- |
| Plasma parameter |  | Group B – Restricted (0-70d)^1^ |  | Group B – Ad-libitum (78-148d) |  | RFI | FL | RFI × FL |
| Trimethylamine N-oxide |  | -0.25 |  | -0.29 |  | 0.30 | 0.008 | 0.43 |
| 1-Methylhistidine |  | 0.00 |  | -0.06 |  | 0.92 | 0.002 | 0.90 |
| 3-Methylhistidine |  | 0.09 |  | 0.11 |  | 0.75 | 0.008 | 0.95 |
| 5-Aminovaleric acid |  | 0.72 |  | 0.74 |  | 0.005* | <0.001* | 0.11 |
| Alpha-amino-butyric acid |  | -0.28 |  | 0.48 |  | 0.55 | 0.09 | 0.06 |
| Asymmetric dimethylarginine |  | 0.01 |  | 0.08 |  | 0.91 | 0.48 | 0.85 |
| Alpha-Aminoadipic acid |  | -0.04 |  | 0.17 |  | 0.72 | 0.57 | 0.38 |
| L-anserine |  | -0.26 |  | -0.58 |  | 0.11 | 1.00 | 0.82 |
| Betaine |  | 0.58 |  | 0.57 |  | 0.04 | <0.001* | 0.27 |
| Carnosine |  | -0.34 |  | -0.39 |  | 0.21 | 0.04 | 0.77 |
| Citrulline |  | 0.42 |  | 0.00 |  | 0.37 | 0.05 | 0.17 |
| Creatinine |  | -0.31 |  | -0.45 |  | 0.21 | 0.002 | 0.66 |
| Cystine |  | -0.04 |  | -0.47 |  | 0.20 | 0.02 | 0.16 |
| Homoarginine |  | 0.51 |  | 0.52 |  | 0.08 | 0.70 | 0.70 |
| Homocysteine |  | 0.60 |  | 0.03 |  | 0.06 | 0.21 | 0.06 |
| Kynurenine |  | 0.15 |  | 0.05 |  | 0.66 | 0.90 | 0.75 |
| Methionine-sulfoxide |  | 0.38 |  | 0.29 |  | 0.34 | 0.12 | 0.87 |
| Ornithine |  | 0.14 |  | -0.16 |  | 0.99 | 0.64 | 0.33 |
| Sarcosine |  | -0.03 |  | 0.73 |  | 0.17 | <0.001* | 0.012 |
| Symmetric dimethylarginine |  | 0.05 |  | -0.03 |  | 0.88 | 0.03 | 0.46 |
| Trans-4-hydroxyproline |  | 0.16 |  | -0.24 |  | 0.82 | 0.76 | 0.18 |
| Alanine |  | -0.17 |  | 0.14 |  | 0.90 | 0.08 | 0.38 |
| Arginine |  | 0.24 |  | 0.11 |  | 0.55 | 0.17 | 0.59 |
| Asparagine |  | 0.01 |  | 0.31 |  | 0.57 | 0.27 | 0.42 |
| Aspartic acid |  | -0.31 |  | 0.35 |  | 0.85 | 0.58 | 0.10 |
| Cysteine |  | 0.18 |  | -0.45 |  | 0.55 | 0.001* | 0.06 |
| Glutamine |  | 0.07 |  | -0.13 |  | 0.93 | 0.005 | 0.44 |
| Glutamic acid |  | -0.20 |  | -0.02 |  | 0.66 | 0.92 | 0.36 |
| Glycine |  | -0.05 |  | 0.46 |  | 0.47 | 0.001* | 0.17 |
| Histidine |  | 0.40 |  | 0.37 |  | 0.12 | 0.03 | 0.90 |
| Isoleucine |  | -0.11 |  | 0.25 |  | 0.87 | 0.006 | 0.32 |
| Leucine |  | 0.01 |  | 0.21 |  | 0.68 | 0.30 | 0.47 |
| Lysine |  | -0.02 |  | 0.36 |  | 0.55 | 0.25 | 0.34 |
| Methionine |  | 0.00 |  | 0.10 |  | 0.87 | 0.007 | 0.66 |
| Phenylalanine |  | -0.29 |  | 0.06 |  | 0.67 | 0.20 | 0.36 |
| Proline |  | 0.06 |  | 0.21 |  | 0.62 | 0.02 | 0.68 |
| Serine |  | 0.52 |  | 0.80 |  | 0.012 | <0.001* | 0.73 |
| Threonine |  | 0.09 |  | -0.25 |  | 0.79 | 0.01 | 0.14 |
| Tryptophan |  | 0.00 |  | -0.07 |  | 0.87 | 0.02 | 0.77 |
| Tyrosine |  | 0.00 |  | 0.05 |  | 0.91 | <0.001* | 0.83 |
| Valine |  | -0.09 |  | 0.01 |  | 0.93 | 0.01 | 0.79 |
| Cholic acid |  | 0.49 |  | 0.23 |  | 0.17 | <0.001* | 0.21 |
| Chenodeoxycholic acid |  | 0.25 |  | 0.54 |  | 0.27 | 0.006 | 0.71 |
| Deoxycholic acid |  | -0.02 |  | 0.24 |  | 0.82 | 0.01 | 0.48 |
| Glycocholic acid |  | 0.02 |  | -0.33 |  | 0.39 | 0.14 | 0.37 |
| Glycochenodeoxycholic acid |  | 0.07 |  | -0.29 |  | 0.69 | 0.04 | 0.30 |
| Glycodeoxycholic acid |  | -0.16 |  | -0.36 |  | 0.24 | 0.04 | 0.54 |
| Glycolithocholic acid |  | -0.05 |  | -0.38 |  | 0.74 | 0.25 | 0.32 |
| Taurocholic acid |  | 0.01 |  | 0.00 |  | 0.28 | 0.10 | 0.26 |
| Taurochenodeoxycholic acid |  | 0.12 |  | -0.37 |  | 0.59 | 0.04 | 0.14 |
| Taurolithocholic acid |  | 0.21 |  | -0.38 |  | 0.14 | 0.27 | 0.39 |
| Beta-alanine |  | -0.48 |  | -0.40 |  | 0.07 | 0.003* | 0.90 |
| Gamma-aminobutyric acid |  | 0.09 |  | 0.52 |  | 0.99 | 0.10 | 0.99 |
| Putrescine |  | 0.02 |  | 0.40 |  | 0.44 | 0.12 | 0.60 |
| Serotonin |  | -0.64 |  | 0.37 |  | 0.16 | 0.80 | 0.004 |
| Dodecanedioic acid |  | 0.29 |  | 0.54 |  | 0.08 | 0.32 | 0.17 |
| Hippuric acid |  | 0.62 |  | -0.28 |  | 0.88 | 0.007 | 0.10 |
| Lactic acid |  | 0.07 |  | 0.05 |  | 0.84 | 0.62 | 0.99 |
| p-cresol sulfate |  | 0.36 |  | -0.13 |  | 0.61 | 0.19 | 0.27 |
| Arachidonic acid |  | -0.18 |  | 0.17 |  | 0.68 | <0.001* | 0.41 |
| Docosahexaenoic acid |  | 0.24 |  | 0.26 |  | 0.37 | <0.001* | 0.69 |
| Eicosapentanoic acid |  | -0.20 |  | 0.00 |  | 0.76 | <0.001* | 0.74 |
| Octadecenoic acid |  | 0.15 |  | 0.42 |  | 0.23 | 0.04 | 0.60 |
| Octadecadienoate |  | 0.18 |  | 0.34 |  | 0.27 | 0.13 | 0.81 |
| Indoleacetic acid |  | 0.41 |  | 0.11 |  | 0.43 | 0.77 | 0.14 |
| Indolepropionic acid |  | 0.20 |  | 0.10 |  | 0.62 | 0.53 | 0.89 |
| Indoxyl sulfate |  | -0.08 |  | 0.04 |  | 0.97 | 0.81 | 0.71 |
| Choline |  | 0.05 |  | 0.48 |  | 0.48 | 0.36 | 0.56 |
| Glucose |  | -0.16 |  | 0.69 |  | 0.69 | 0.09 | 0.13 |
| Non-esterified fatty acids |  | 0.06 |  | 0.46 |  | 0.36 | 0.05 | 0.47 |
| Beta-hydroxyburytrate |  | 0.26 |  | 0.04 |  | 0.80 | 0.001* | 0.57 |
| Urea |  | 0.48 |  | 0.21 |  | 0.30 | 0.44 | 0.44 |
| Alanine aminotransferase |  | -0.22 |  | -0.24 |  | 0.40 | 0.002* | 0.59 |
| Aspartate aminotransferase |  | -0.06 |  | -0.05 |  | 0.84 | 0.23 | 0.98 |
| Alkaline phosphatase |  | 0.27 |  | 0.13 |  | 0.51 | 0.002 | 0.63 |
| Gamma-glutamyl transferase |  | 0.27 |  | 0.28 |  | 0.34 | 0.79 | 0.70 |
| Natural abundance of ^15^N |  | 0.29 |  | 0.20 |  | 0.34 | <0.001* | 0.72 |
| Insulin |  | 0.26 |  | 0.07 |  | 0.46 | 0.21 | 0.67 |
| IGF-1 |  | -0.41 |  | 0.34 |  | 0.78 | 0.08 | 0.11 |

*P values with a star are also FDR <0.05

^1^ Animals in the first RFI test were the same as those in the second RFI test and maintained the same RFI values (ranging from -0.98 to 0.61 kg/d) observed ad libitum.
